# The pathophysiological basis and consequences of fever

**DOI:** 10.1186/s13054-016-1375-5

**Published:** 2016-07-14

**Authors:** Edward James Walter, Sameer Hanna-Jumma, Mike Carraretto, Lui Forni

**Affiliations:** Department of Intensive Care Medicine, Royal Surrey County Hospital, Egerton Road, Guildford, Surrey GU2 7XX UK

**Keywords:** Hyperthermia, Fever, Organ failure, Physiopathology, Heatstroke

## Abstract

There are numerous causes of a raised core temperature. A fever occurring in sepsis may be associated with a survival benefit. However, this is not the case for non-infective triggers. Where heat generation exceeds heat loss and the core temperature rises above that set by the hypothalamus, a combination of cellular, local, organ-specific, and systemic effects occurs and puts the individual at risk of both short-term and long-term dysfunction which, if severe or sustained, may lead to death. This narrative review is part of a series that will outline the pathophysiology of pyrogenic and non-pyrogenic fever, concentrating primarily on the pathophysiology of non-septic causes.

## Background

*“Humanity has but three great enemies: fever, famine, and war, and of these by far the greatest, by far the most terrible, is fever.” (William Osler)*

The normal human temperature is considered to be 37 °C, but may vary by up to 1 °C in healthy individuals [1]. Elevated core temperature is a common finding in intensive care, affecting up to 70 % of patients [2]. Despite the general usage of the terms ‘pyrexia’, ‘fever’, and ‘hyperthermia’, they are not yet universally defined. The American College of Critical Care Medicine, the International Statistical Classification of Diseases, and the Infectious Diseases Society of America define fever as a core temperature of 38.3 °C or higher, i.e. just above the upper limit of a normal human temperature, irrespective of the cause [1]. Fever has its etymological basis in Latin, meaning simply ‘heat’, and pyrexia comes from the Greek ‘pyr’, meaning fire or fever. Some sources use the terms interchangeably, whereas others preserve ‘fever’ to mean a raised temperature caused by the action of thermoregulatory pyrogens on the hypothalamus; for instance, in sepsis and inflammatory conditions [3].

Hyperthermia also has no agreed definition; it has been defined as a core temperature above 38.2 °C, irrespective of the cause [3]. Others use it for the classification of those conditions that increase the body’s temperature above that set by the hypothalamus, and therefore specifically exclude those where fever is caused by pyrogens [4], being due to heat exposure or unregulated heat production in excess of heat loss. Common causes include classical and exertional heatstroke, and drug-related illnesses (for example, malignant hyperthermia and neuroleptic syndrome).

There is, however, increasing evidence that many conditions considered non-pyrogenic may stimulate an inflammatory response, and the division into pyrogenic and non-pyrogenic may therefore be less clear-cut than previously understood.

## Generation of fever

Sepsis accounts for up to 74 % of fever in hospitalised patients [5] and, of the remainder, malignancy, tissue ischaemia, and drug reactions account for the majority [6]. Neurogenic fever, and fevers associated with endocrinopathy, are rarer.

### Sepsis

**Fig. 1:**
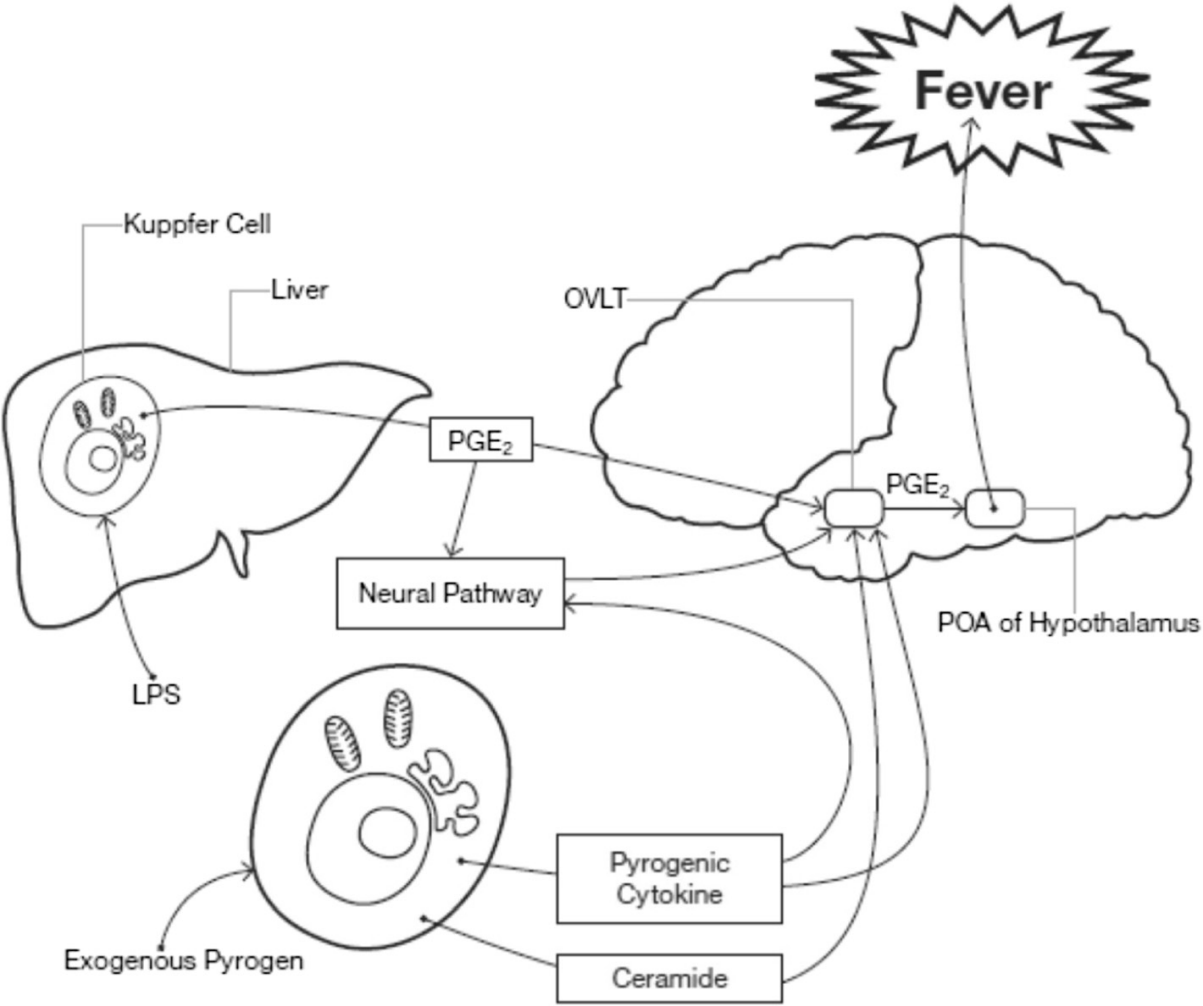
Proposed mechanisms for the generation of fever in sepsis. Stimulation of sentinel cells by exogenous pyrogens produces endogenous pyrogens which stimulate fever production in the pre-optic area (*POA*) of the hypothalamus by the second messengers prostaglandin E_2_ (*PGE*_*2*_), and ceramide. PGE_2_ is also produced from Kupffer cells in the liver in response to stimulation from lipopolysaccharide (*LPS*), which additionally stimulates the POA via the vagus nerve. *OVLT* organum vasculosum of the lamina terminalis

Pyrogenic fever is a common response to sepsis in critically ill patients, and the generation of fever occurs through several mechanisms. The interaction of exogenous pyrogens (e.g. micro-organisms) or endogenous pyrogens (e.g. interleukin (IL)-1, IL-6, tumour necrosis factor (TNF)-α) with the organum vasculosum of the lamina terminalis (OVLT) leads to the production of fever. Exogenous pyrogens may stimulate cytokine production, or may act directly on the OVLT. The OVLT is one of seven predominantly cellular structures in the anterior hypothalamus within the lamina terminalis, located in the optic recess at the anteroventral end of the third ventricle. Being a circumventricular organ it is highly vascular and lacks a blood–brain barrier (BBB), permitting it to be stimulated directly by pyrogenic substances. Its stimulation leads to increased synthesis of prostanoids including prostaglandin (PG)E_2_, which acts in the pre-optic nucleus of the hypothalamus slowing the firing rate of the warm sensitive neurons and resulting in an increase in body temperature. The bioactive lipid derivative, ceramide, which has a proapoptotic as well as a cell signalling role, may act as a second messenger independent of PGE_2_, and may be of particular importance in the early stages of fever generation [7]. Lipopolysaccharides (LPS) from gram-negative bacteria may stimulate peripheral production of PGE_2_ from hepatic Kupffer cells [8, 9]. LPS-stimulated fever may also be neurally mediated [10]. Neural pathways may account for the rapid onset of fever, with cytokine production responsible for the maintenance, rather than the initiation, of fever [11]. Fever generation is also thought to occur by signalling via the Toll-like receptor cascade, which may be independent of the cytokine cascade [12] (Fig. 1).

The febrile response is well preserved across the animal kingdom, with some experimental evidence suggesting it may be a beneficial response to infection. Retrospective data analysis shows that a raised temperature in patients with infection in the first 24 h following admission to the intensive care unit (ICU) is associated with a better outcome compared with normothermia or hyperthermia above 40 °C [13], and that a temperature between 37.5 °C and 39.4 °C trends towards improved outcome compared with normothermia [14]. In elderly patients with community-acquired pneumonia, the observed mortality rate was significantly higher in patients who lacked fever (29 %) when compared with patients who developed a febrile response (4 %) [15]. A temperature greater than 38.2 °C has also been found to have a protective role against invasive fungal infections in the ICU [16]. The raised temperature may provide protection by several mechanisms. Firstly, human infective pathogens often demonstrate optimal replication at temperatures below 37 °C; thus an elevated host temperature inhibits reproduction [17]. Secondly, increasing the temperature in vitro from 35 °C to 41.5 °C increases the antimicrobial activity of many classes of antibiotics [18]. Thirdly, a rise in temperature may also be associated with an increase in innate immunity associated with microbial destruction [19]. Interestingly, at temperatures above around 40 °C there is a further mortality increase [13, 14], suggesting that at this stage the deleterious effects of hyperthermia on organ and cellular function outweigh any benefit conferred from hyperpyrexia in acute sepsis. These potential benefits of fever in sepsis may not be well recognised; in one survey of fever monitoring in sepsis from UK ICUs, 76 % of ICU physicians would be concerned about a temperature of 38–39 °C, and 66 % would initiate active cooling at that point [20].

In contrast with a fever in response to sepsis, a non-pyrogenic fever is not of any perceived teleological benefit. A temperature of 37.5 °C or greater at any point during an ICU admission trends towards a worse outcome, and becomes significant at temperatures greater than 38.5 °C [14].

### Fever associated with inflammation

In critically ill patients, inflammation is commonly observed to aid repair after traumatic or infective insults. The four cardinal features of pain, heat, redness, and swelling were originally described by Celsus around 2000 years ago and, at about the same time, Hippocrates noted that the fever was of benefit. Fever is a ubiquitous component of inflammation across the animal kingdom, and enhances the host response. A large number of both the cell-derived and plasma-derived inflammatory mediators are pyrogenic; fever associated with inflammation is probably mediated in a similar way to sepsis as described above. Chronic inflammation is deleterious; the recently described compensatory anti-inflammatory response syndrome (CARS) restores homeostasis, and it is likely that the magnitude and relative timings of the inflammatory and anti-inflammatory responses are both important in determining the host outcome.

Fever in patients with malignancy is reported to be sepsis related in around two thirds of cases [21]. The tumour is the direct cause of fever in less than 10 % of febrile episodes; tumour necrosis and production of pyrogenic cytokines is the likely pathogenesis [21].

Regulated autoimmunity is considered to be a natural physiological reaction; however, pathological autoimmunity occurs because of higher titres of more antigen-specific antibodies, often of the IgG isoform, and a reduction in self-tolerance. There are five pathogenic processes associated with autoimmune disease development, and in excess of 80 diseases have been described; fever is considered to be cytokine mediated in the majority of cases [22].

Autoinflammatory conditions differ from autoimmune diseases. In the former, the innate immune system directly causes inflammation without a significant T-cell response, whereas in the latter the innate immune system activates the adaptive immune system, which is in itself responsible for the inflammatory process. The former are also known as periodic fever syndromes, highlighting the intermittent febrile nature of these conditions. Examples include familial Mediterranean fever and some arthopathies, including adult-onset Still’s disease. Most autoinflammatory conditions are genetic, and a large number are related to abnormalities in pro-inflammatory cytokine handling, for example IL-1 or interferon (IFN) signalling, or constitutive NF-kB activation, offering therapeutic targets.

### Drug-induced fever

The causes of drug-induced fever are shown in Table 1 [23]. Pharmacological agents may cause fever by a number of pathophysiological mechanisms. These include interference with the physiological mechanisms of heat loss from the peripheries, interference with central temperature regulation, direct damage to tissues, stimulation of an immune response, or pyrogenic properties of the drug.

**Table 1:** Causes of drug-induced hyperthermia

Class	Examples of causes
Antimicrobial agents	β-lactam antibiotics (piperacillin, cefotaxime) Sulphonamides
Malignant hyperthermia	Suxamethonium Volatile anaesthetic agents
Neuroleptic malignant syndrome	Dopamine antagonists (chlorpromazine, haloperidol) Atypical agents (serotonin and dopamine antagonists) (olanzapine, risperidone, paliperidone, aripiprazole, quetiapine)
Serotonin syndrome	Antidepressants (monoamine oxidase inhibitors, tricyclic antidepressants, selective serotonin reuptake inhibitors, serotonin noradrenaline reuptake inhibitors, bupropion) Opioids (tramadol, pethidine, fentanyl, pentazocine, buprenorphine oxycodone, hydrocodone) Central nervous system stimulants (MDMA, amphetamines, sibutramine, methylphenidate, methamphetamine, cocaine) Psychedelics (5-methoxy-diisopropyltryptamine, lysergide) Herbs (St John’s Wort, Syrian rue, Panax ginseng, nutmeg, yohimbine) Others (tryptophan, l-dopa, valproate, buspirone, lithium, linezolid, chlorpheniramine, risperidone, olanzapine, antiemetics (ondansetron, granisetron, metoclopramide), ritonavir, sumatriptan)
Propofol infusion syndrome	Propofol
Anticholinergic agents	Anticholinergics (atropine, glycopyrrolate), Antihistamines (chlorpheniramine), Antipsychotics (olanzapine, quetiapine), Antispasmodics (oxybutynin), Cyclic antidepressants (amitriptyline, doxepin) Mydriatics (tropicamide)
Sympathimometic agents	Prescription drugs (e.g. bronchodilators) Non-prescription drugs (e.g. ephedrine in cold remedies) Illegal street drugs (e.g. cocaine, amphetamines, methamphetamine (‘ecstasy’), mephedrone) Dietary supplements (e.g. ephedra alkaloids)
Piperazine compounds	Anti-emetic (cyclizine) Anti-helminths Legal ‘club drugs’ (‘Legal X’, ‘Legal E’, ‘Frenzy’)
Synthetic cathinones	Street drugs (mephedrone, ‘meow-meow’) Bupropion (anti-depressant and anti-smoking agent)

Taken from [23] with permission

A common mechanism in many of these drugs is considered to be stimulation of non-shivering thermogenesis (NST), primarily in brown adipose tissue and skeletal muscle. Under normal conditions, cellular oxidative phosphorylation allows the synthesis of ATP from ADP for cellular metabolism. NST uncouples the proton movement from this pathway, allowing the energy to be dissipated as heat, under the control of uncoupling proteins, ultimately influenced by thyroid hormones and catecholamines. A number of agents, including sympathomimetics and those which act via the serotonin pathway, are thought to cause fever by modifying the NST pathway at a central, peripheral, or cellular level [24].

### Fever after brain injury

Fever after acute brain damage, from trauma or a vascular event, is common, and is independently associated with a worse outcome. The mechanism of fever generation is probably multi-factorial; 41 % of deaths after traumatic brain injury (TBI) in one series displayed hypothalamic lesions, suggesting thermal dysregulation in some cases [25]. Alterations in cellular metabolism, a shift to anaerobic metabolism, and ischaemic–reperfusion injury are all associated with thermogenesis [26]. The cerebral production of a large number of inflammatory and pyrogenic cytokines is increased acutely [27]; IL-6 in particular is associated with fever production after a stroke, and with a worse outcome. After cerebral haemorrhage, both the presence of blood and the presence of its degradation products are associated with heat production [28]. Recent work suggests a protective role for uncoupling of mitochondrial oxidative phosphorylation following neurotrauma under the regulation of uncoupling proteins [29]; the dissipation of the proton gradient produces heat.

Brain injury following a cardiac arrest is well recognised, but the pathology is complex and probably involves multiple mechanisms, including cell death, excitotoxicity, cell signalling changes, ischaemia–reperfusion, and alterations in cellular metabolism [30]; this is very similar to those described following brain injury from other causes, and, as such, the mechanisms of thermogenesis are likely to be similar. The teleological benefit of pyrexia following brain injury is uncertain.

### Endocrine fever

Thyroid hormones are essential for regulation of energy metabolism. Hyperthyroidism is associated with hyperthermia; patients with thyroid storm have an average body temperature of 38.0 °C; temperatures above 41 °C have been reported [31]. The mechanism of thermogenesis is not clear; the classical view is that metabolism of peripheral tissues increases through a peripherally mediated pathway. Recent work suggests that thyroid hormones may instead act centrally to increase the hypothalamic ‘set-point’, and that centrally driven neurogenic activation of uncoupling protein-1 acting on brown adipose tissue may instead be responsible for the thermogenesis [32]. The converse relationship is also present: levels of serum T3, even in non-thyropathic individuals, decrease with increasing body temperature and, above 40 °C, T3 levels would be consistent with severe hypothyroidism. The levels of T4 and thyroid-stimulating hormone (TSH) are unchanged with changes in body temperature [33].

Adrenal insufficiency is rarely associated with fever, but the hyperthermia may be related to the underlying pathology; autoimmunity accounts for the majority of primary insufficiency. A malignant process, or an infectious process, account for a proportion of the remainder; all of the patients in the original description had adrenal tuberculosis [34].

A fever has been reported in 28 % of patients hospitalised with a pheochromocytoma [35]; a large tumour, the presence of necrosis, and higher metabolite excretion increase the likelihood of pyrexia [35].

## Mechanisms of damage from fever

**Fig. 2:**
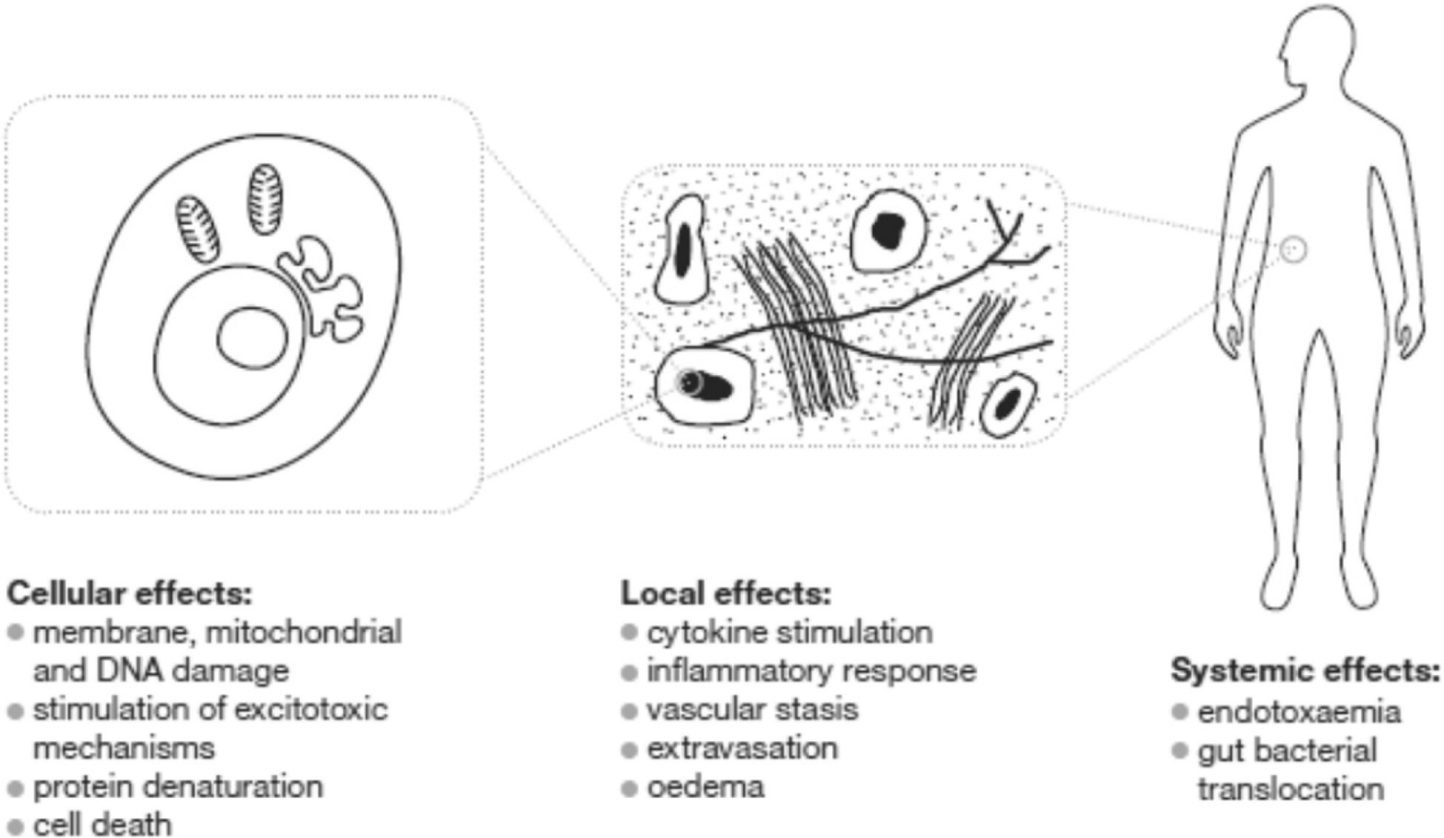
Diagrammatic representation of the mechanisms of damage from hyperthermia

There are a number of pathophysiological mechanisms for the deleterious effects of a fever, classified as follows (Fig. 2):

- Direct cellular damage
- Local effects, e.g. stimulation of cytokines and inflammatory response
- Systemic effects, e.g. gut bacterial translocation

### Cellular damage

Hyperthermia is directly cytotoxic, affecting membrane stability and transmembrane transport protein function. Consequently, ionic transport is disrupted leading to increased intracellular sodium and calcium with a reduced intracellular potassium concentration. Protein and DNA synthesis is disrupted at various stages in the pathway; while RNA and protein synthesis may recover quickly after cessation of hyperthermia, DNA synthesis remains disrupted for longer [36]. The nuclear matrix shows damage at lower temperatures than other parts of the cell, with significant endothermic changes observed at 40 °C [37]. Direct cell death in humans occurs at temperatures of around 41 °C, with the rate of cell death increasing markedly with even modest further increases in temperature [36, 38]. The thermal energy required for cell death is similar to that required for protein denaturation, suggesting that hyperthermic cell death may occur primarily through its effect on protein structure, although cell death occurs primarily through necrosis or from apoptosis depending on the cell line and the temperature [36]. Cells in mitosis are more thermosensitive than cells in other phases of replication. Given that organ dysfunction occurs at temperatures lower than that required for in-vitro cell death, milder degrees of hyperthermia are also likely to affect cell structure and function with a degree of reversibility.

### Local effects

#### Effect of cytokines and the inflammatory response

The role of cytokines in heat stress is unclear, with an inconsistent response to thermal stress. The levels of a number of pro-inflammatory and anti-inflammatory cytokines are elevated at the time of hyperthermia from heatstroke. Acute phase reactants may also increase. Of these, some (for example, INFγ, IL-1β) are raised in a proportion of patients, whereas IL-6 may be elevated in all patients [39]. Furthermore, there is some correlation with outcome; the rise in IL-6 and the duration of the increased expression is related to mortality, independent of the maximum core temperature obtained [40]. Mice pre-treated with IL-6 before exposure to heat take longer to reach 42.4 °C, showing less organ damage, and attenuation in the increase of other cytokines [41]. Antagonism of IL-1 also improves survival [42].

The cytokine profile of the two forms of heatstroke, classical and exertional, show similarities, and mirrors that produced by exercise [43]. The profile also shows similarities to that produced by endotoxaemia, which is considered to be of importance in the cytokine expression—abolition of endotoxaemia significantly reduces cytokine production [43].

Development of other hyperthermic states may also be associated with inflammatory mediators. Neuroleptic malignant syndrome (NMS) may be at least partly driven by an acute phase response; acute phase response mediators are reported to rise, and peak at 72 h. Conversely, levels of anti-inflammatory agents such as serum iron and albumin initially decline then return to the normal range, coinciding with clinical improvement [44]. It is proposed that the acute phase response may be triggered by the heat stress per se, or by muscle breakdown, or by interaction between a virus and the drug, or the immune system [45]. IL-6 and TNFα levels have also been found to be significantly increased in NMS [46], as has IL-6 in malignant hyperthermia (MH) [47].

#### Protection by heat shock proteins

Heat shock proteins (HSP) are a family of cell-derived proteins that offer protection against a range of insults, including heat. They are expressed in response to the insult, and their effect may depend on their location. Intracellularly located HSPs have a protective role, including correcting misfolded proteins, preventing protein aggregation, transport of proteins, and supporting antigen processing and presentation, and limiting apoptosis. In contrast, membrane-bound or extracellular HSPs may be immunostimulatory, and appear to induce cytokine release or provide recognition sites for natural killer cells. HSPs may also have both pro-apoptotic and anti-apoptotic actions [48, 49].

#### Vascular changes

Animal studies suggest that changes to the vasculature occur rapidly after the onset of hyperthermia and, while some organs are more tolerant to heat stress than others, the majority of organs show similar changes consisting of capillary dilatation, vascular stasis, and extravasation into the interstitium, observed after 30 min at 40.5 °C [50].

### Systemic effects

#### Gastrointestinal bacterial and endotoxin translocation

Non-pyrogenic hyperthermia increases gut bacterial translocation and the gastrointestinal (GI) tract and BBB appear to be more permeable to toxins than during normothermia [51, 52]. Bacterial and endotoxin translocation are also implicated in the development of multi-organ dysfunction in non-pyrogenic hyperthermia. For example, antibiotic administration to dogs with heatstroke appears to improve their survival, suggesting that bacteraemia may have a role even in non-pyrogenic conditions [53]. In a similar study, raising the core temperature in monkeys from 37.5 °C to 39.5 °C and then up to 44.5 °C increased plasma LPS concentration. In the animals pre-treated with oral kanamycin, which is very poorly absorbed, and heated to 44.5 °C, no increase in plasma LPS concentrations were seen and there was improved haemodynamic stability, suggesting that the plasma LPS originated from the GI tract [54]. Epidemiological studies after classical heatstroke have demonstrated that over 50 % of heatstroke patients show evidence of concomitant bacterial infections [55]. Furthermore, procalcitonin, which has a high sensitivity and specificity for detecting bacteraemia, was elevated in 58 % of patients with classical heatstroke, which was associated with mortality [56]. However, microbiological and clinical evidence of infection was not significantly higher in this group, and therefore it is unclear whether this represents undiagnosed bacteraemia or procalcitonin elevated in the absence of infection.

### Genetics

Genotypic and phenotypic differences may account for how tolerant a particular individual is to heat exposure. Individuals who demonstrate heat-intolerance may show a reduction in HSP levels and, in addition, their vasculature may be less reactive to heat stress [57]. Well-described genotypic differences are seen in particular conditions. MH affects up to 1 in 5000 patients, and is more common in males and in young people, although it can affect all age groups including neonates [58]. It has also been observed in other species, such as dogs, cats, horses and pigs. Mutation in the ryanodine receptor (RYR) accounts for up to 70 % of cases, with more recent genetic abnormalities also having been identified [59]. RYRs in the sarcoplasmic reticulum of skeletal muscle form calcium channels and are the main mediators of calcium-induced calcium release in animal cells. In MH, the RYR functions abnormally such that calcium is released in a greater than normal amount and heat is generated during the processing of this excess calcium. The first documented survivor of MH was in Australia in 1961; a young man required surgery for a fractured tibia. Ten of his family members had previously developed uncontrolled hyperthermia and died during general anaesthesia with ether [60].

Exertional heatstroke (EHS) is increasingly observed in endurance athletes [61]. EHS has clinical and biochemical similarities to MH, and there are case reports of patients with both conditions. While some patients with EHS display mutations in the RYR1 gene, the genetics probably differ from MH, although some authorities advise that heatstroke patients should go on to be tested for MH as they may be susceptible to its development [62]. Recently, there has been some interest in another similar sarcoplasmic skeletal muscle protein, calsequestrin (CASQ1), which appears to modulate the function of RYR1. Ablation of CASQ1 in mice increases the risk of MH-like episodes when exposed to both heat and halothane, supporting the possibility that there is a genetic basis to EHS similar to that of MH [63].

Other hyperthermic states may also have a genetic basis. Genetic mutations or polymorphisms in the dopamine D2 receptor, serotonin receptor, and cytochrome P450 2D6 have been studied in cases of NMS [64]. Such cases may run in families, suggesting a genetic mechanism for predisposition to the syndrome. In a study of patients who had developed NMS, the frequency of the A1 allele of the DA2 receptor was significantly higher in the patients who developed NMS (56.8 %) than in the control group of patients with schizophrenia who had not (35.1 %). The proportion of patients who were A1 carriers was significantly higher in the patients with NMS compared with those without (93.3 % vs 57.2 %) [65]. However, the relationship between NMS and serotonin receptor mutations remains currently undetermined. Early work in patients who are genetically deficient in the cytochrome P450 2D6 enzyme suggests that they may be more susceptible to the effects of serotonin-containing drugs [66].

EHS is more common in men than women; whether this is the protective effect of oestrogen, or the reduced muscled muscle bulk in women compared with men, or genetic differences is not clear.

## Deleterious consequences of pyrexia

Most patients fully recover after a period of hyperthermia, but patients exposed to higher temperatures and for longer periods of time are more at risk of complications, which may lead to multi-organ failure and death in extreme cases. The similarities between the different hyperthermic aetiologies suggest that the pathological features are at least partly a result of hyperthermia, irrespective of the cause.

The risk from hyperthermia may be significant; heatstroke is the most severe form of heat illness with a mortality rate of up to 58 % [67] to 64 % [68]. Classical heatstroke, often seen in meteorological heat waves, is responsible for thousands of excess deaths each year. Most survivors appear to recover fully, but there is increasing concern over long-term organ dysfunction, susceptibility to further injury, and delayed mortality.

Immediate cooling remains the mainstay of treatment, a delay in a reduction in the temperature being associated with increased mortality [68]. In classical heatstroke, cooling to below 38.9 °C within 60 min is associated with a trend towards improved survival [69]. Hyperthermia is associated with the inflammatory cascade [43]; heatstroke in particular is considered a pro-inflammatory and pro-coagulant condition. Given this, steroids [70], mannitol [70], and recombinant activated protein C [71, 72] have all been studied as putative treatments, and have shown benefit in trials; however, none are currently recommended for clinical practice. Anti-pyretic drugs would not be expected to have a significant effect in non-pyrogenic hyperthermia and, although non-steroidal anti-inflammatory drugs (NSAIDs) have not been extensively studied, aspirin may have beneficial effects on survival in animal studies [73]. Neither aspirin nor paracetamol have been shown to be of any proven benefit in humans and are therefore not recommended in temperature control in heatstroke.

### Specific organ dysfunction

Hyperthermia has many systemic effects, which may present as specific organ dysfunction.

#### Gastrointestinal tract

Systemic hyperthermia increases the permeability of the GI tract, and increases the rate of gut bacterial translocation. Blood flow to the GI tract is reduced at temperatures above 40 °C [74] and hyperthermia damages cell membranes, denatures proteins, and may increase oxidative stress. This leads to loss of the GI barrier integrity and increases the potential for endotoxaemia, which initiates release of pro-inflammatory cytokines leading to a systemic inflammatory cascade [51]. GI oedema and petechial haemorrhage are also described [75].

A theoretical mechanism following hyperthermia to the GI tract appears to be increased free radical production from the splanchnic viscera, which may stimulate oxidative stress and contribute to cellular dysfunction [74]. Free radical production can be increased in the presence of heavy metals and this may exacerbate cytotoxicity. Heavy metals themselves may also translocate across a dysfunctional BBB, and are implicated in the development of hyperthermia-induced neurocognitive dysfunction [76].

#### Renal

The glomerular filtration rate reduces after an increase of 2 °C, and worsens further with increasing temperature. Plasma concentrations of creatinine and urea consequently increase [77]. Morphological studies demonstrate glomerular capillary dilatation, haemorrhage into the interstitium, and vascular stasis, in small and large vessels [50]. Stimulation of the renin–angiotensin system in hyperthermia reduces renal blood flow [78]. Direct thermal injury, renal hypoperfusion, and rhabdomyolysis also probably contribute to acute kidney injury (AKI).

The development of EHS (>40 °C) in endurance athletes significantly increases the risk of AKI compared with those without EHS. Military data suggest that one in six hospitalised EHS victims will develop AKI [79] in comparison with marathon runners generally; the Comrades marathon have reported an average of only one runner each year admitted with renal failure [80].

Classical heatstroke is also associated with the development of AKI; for example, of 22 patients admitted to an ICU after heatstroke during a heatwave, serum creatinine levels were significantly higher 24 h after admission, and 18 % required renal replacement therapy (RRT). The degree of renal impairment was worse in non-survivors than in those who survived [68]. Of 58 patients hospitalised with classical heatstroke during the 1995 Chicago heat wave, 53 % had at least moderate renal impairment [55].

AKI has been reported in one series of patients with neuroleptic malignant syndrome to occur in 7 out of 24 (30 %) patients, of whom 2 (8 %) required RRT [81]. Renal failure sufficient to require RRT has also been described after hyperthermia due to NMS [82], MH [83] and recreational drug use [84].

#### Cardiovascular system

In the acute phase, patients tend to be hypotensive, with a hyperdynamic circulation and a high cardiac output. The hypotension is probably a combination of redistribution of blood, and nitric oxide-induced vasodilatation. The electrocardiogram in heatstroke and MH may show a variety of abnormalities, including conduction defects, QT and ST changes, T-wave abnormalities, and malignant arrhythmias [85]. In addition, cardiac dysfunction and associated pulmonary oedema have also been described [86].

In common with other organs, myocardial vessels are dilated, and extravasation occurs into the myofibril structure. Fragmentation of the myocardial fibres occurs [50]. Serum troponin I levels are significantly raised and, interestingly, more so in non-survivors [68]. Whether this represents myocardial cytotoxicity, myocardial disruption, or another problem is not currently clear.

#### Brain

Neurological and cognitive dysfunction may occur acutely after an episode of hyperthermia and may lead to chronic damage, reported to occur in 50 % of survivors discharged from an ICU after heatstroke [87]. The pathophysiological mechanisms are presumed to be similar to those described above, but, in addition, the integrity of the BBB is disrupted allowing translocation of systemic toxins to enter the cerebral circulation. If neurological symptoms fail to improve after the acute episode, cerebellar dysfunction predominates. This is thought to be a result of the sensitivity of the Purkinje cells to thermal damage.

#### Liver failure

Liver dysfunction is common. At temperatures above 40 °C, elevations in plasma aspartate transaminase (AST) and alanine transaminase (ALT) are observed [88] and the hepatocellular damage has been sufficient to require transplant in some cases; however, results from transplantation are disappointing, with only a minority surviving long-term [89]. Hence, conservative management has been advocated in patients who would otherwise meet the criteria for transplantation [89].

Similar to histological changes in other organs, small and large vessel dilatation is seen, with stasis and haemorrhage [50]. A reduction in liver blood flow is also implicated [90]. Liver dysfunction may continue to deteriorate even after cessation of the hyperthermia [68].

#### Haemostatic system

Coagulopathy is common, with a reported incidence of 45 % in classical heatstroke [55], and probably contributes to the multi-organ dysfunction in hyperthermia. Thrombocytopenia, increased plasma fibrin degradation products, prolonged clotting times, and spontaneous bleeding are often seen. This probably reflects hepatic dysfunction, as coagulopathy is rare without liver derangement and is temporally related to alterations in liver function [91]. Hyperthermia inhibits platelet aggregation, which becomes increasingly marked at higher temperatures, and may begin to happen at 38 °C [92]. Disseminated intravascular coagulation (DIC) may also be driven by release of pro-coagulant cellular components from damaged muscle.

### Long-term follow-up

Even in survivors of the acute episode, hyperthermia reduces life expectancy and worsens functional outcome. In one epidemiological study of patients with classical heatstroke, the 28-day mortality was 58 %, increasing to 71 % at 2 years [67]. An episode of exertional heatstroke is associated with an increased risk of mortality of 40 % after recovery from the initial episode [93].

Heatstroke is reported to cause moderate to severe functional impairment in 33 % of survivors at 1 year [55], with 41 % of survivors requiring institutional care at 1 year [66]. There may be little or no improvement after discharge from hospital [55].

## Conclusions

A mild elevation in core temperature is of benefit in sepsis. Non-pyrogenic hyperthermia is associated with short-term, medium-term, and long-term effects in a variety of organs. The damage occurs via a number of local and systemic mechanisms. Additionally, there appears to be emerging evidence of an overlap in the mechanisms of heat generation in different conditions. The evidence is that in sepsis the beneficial effects of pyrexia may balance these deleterious factors. However, in non-sepsis, the accumulation of the deleterious consequences of hyperthermia occurs early, at even mild degrees of fever. Hyperthermia above 40 °C appears to carry a high mortality by whatever cause. Early recognition, immediate cooling, and organ support are the mainstays of treatment, and to this end an improved understanding of the pathophysiology will continue to develop.

## Abbreviations

AKI, acute kidney injury; BBB, blood–brain barrier; CASQ1, calsequestrin; EHS, exertional heatstroke; GI, gastrointestinal; HSP, heat shock proteins; ICU, intensive care unit; IFN, interferon; IL, interleukin; LPS, lipopolysaccharides; MH, malignant hyperthermia; NMS, neuroleptic malignant syndrome; NSAID, non-steroidal anti-inflammatory drug; NST, non-shivering thermogenesis; OVLT, organum vasculosum of the lamina terminalis; PG, prostaglandin; RRT, renal replacement therapy; RYR, ryanodine receptor; TNF, tumour necrosis factor
